# Supplementary material for: Systematic review and narrative synthesis of the experiences of individuals with chronic pain participating in digital pain management interventions
Source: PLoS One. 2024 Jul 8;19(7):e0306455. doi: 10.1371/journal.pone.0306455 (PMC11230547; doi:10.1371/journal.pone.0306455)
Supplement: Attachment — Submitted filename: JS ES LW Systematic Review Response to Reviewers.pdf [file pone.0306455.s003.pdf]

Southern Health
NHS Foundation Trust

Musculoskeletal and Pain Services
New Forest Enterprise Centre
Rushington Business Park
Chapel Lane
Totton SO40 9LA
justin.strain@southernhealth.nhs.uk

Dr M Kajbafvala
Academic Editor
PLOS ONE

7th June 2024

Dear Dr Kajbafvala,

Re our manuscript: "Systematic Review and Narrative Synthesis of the experiences of individuals with chronic pain participating in digital pain management interventions".

Thank you for giving us the opportunity to revise our manuscript and we are pleased that you found our article interesting. Please see our following responses to your reviewer's comments outlining how we have addressed the several suggestions you have indicated for further improving our article.

***1**. Please ensure that your manuscript meets PLOS ONE's style requirements, including those for file naming. The PLOS ONE style templates can be found at https://journals.plos.org/plosone/s/file?id=wjVg/PLOSOne_formatting_sample_main_body.pdf https://journals.plos.org/plosone/s/file?id=ba62/PLOSOne_formatting_sample_title_authorsaffiliations.pdf*

File naming style requirements have now been met – this is only applicable to Fig1 and Fig2.

***2. Funding Statement:** Thank you for stating the following financial disclosure:"JS received a Research Internship and Research Initiation Award to provide funded time for this research. This included academic supervision from ES and LW. These awards were both funded by NIHR Applied Research Collaboration (ARC) Wessex (https://www.arc-wx.nihr.ac.uk/) and NHS England (https://www.england.nhs.uk/). "Please state what role the funders took in the study. If the funders had no role, please state: "The funders had no role in study design, data collection and analysis, decision to publish, or preparation of the manuscript." If this statement is not correct you must amend it as needed. Please include this amended Role of Funder statement in your cover letter; we will change the online submission form on your behalf.*

We have added the following to the funding statement: 'ES is supported by NIHR ARC Wessex'. I confirm that the funders had no role in study design, data collection and analysis, decision to publish, or preparation of the manuscript.

***3. Data Availability Statement:** Please confirm at this time whether or not your submission contains all raw data required to replicate the results of your study.*

I confirm that all relevant data are within the manuscript.

Southern Health
NHS Foundation Trust

4. Supporting Files: *Please include captions for your Supporting Information files at the end of your manuscript.*

Other than 2 figures which appear within the main manuscript (Fig1 and Fig2) there are no additional Supporting Files. Fig1 and Fig2 have been uploaded to PACE and have met PLOS requirements.

5. Reference List: *Please review your reference list to ensure that it is complete and correct.*
References have been rewritten as numbered references to comply with the journal's style requirements.

6. Abstract: *The abstract section should be written according to the guidelines of the journal (Background, Methods, Results, Conclusion). Please write keywords based on MeSH terms.*

The abstract has now been written in accordance with the journal guidelines. MeSH terms: Pain Management, Chronic Pain and Qualitative Research have also been added.

7. Methodology: *In the " search strategy " section, Google Scholar is deleted; Because it is not a database but a search engine.*

Google Scholar has now been deleted from the search strategy section but kept in description of citation tracking.

8. Discussion: *I suggest you explain more how your study will help the health system and patients.*

We thank the editor/reviewer for this comment. We have now added further detail to the discussion on how our study will help the health system and patients.

Thank you again for giving us the opportunity to revise our manuscript. We look forward to hearing back from you at your earliest convenience.

Yours sincerely.

Justin Strain, and on behalf of my other authors.

Justin Strain (He/ Him)
Consultant Musculoskeletal Allied Health Professional
Clinical Specialist Physiotherapist in Pain Management and Spines
Southern Health NHS Foundation Trust

Justin.Strain@southernhealth.nhs.uk

OUR VALUES
People first Partnership Respect
