# Supplementary material for: Systematic review and narrative synthesis of the experiences of individuals with chronic pain participating in digital pain management interventions
Source: PLoS One. 2024 Jul 8;19(7):e0306455. doi: 10.1371/journal.pone.0306455 (PMC11230547; doi:10.1371/journal.pone.0306455)
Supplement: Attachment — Submitted filename: renamed_8683a.docx [file pone.0306455.s002.docx]

**Dear Authors**,

Thank you very much for your interesting article that discusses an important topic. The title of your article " *Systematic Review and Narrative Synthesis of the experiences of individuals with chronic pain participating in digital pain management interventions* " is an interesting idea and I enjoyed reading it. However, I have several suggestions for improving your article.

**FIRST;** *Please ensure that your manuscript meets PLOS ONE's style requirements, including those for file naming. The PLOS ONE style templates can be found at*

[*https://journals.plos.org/plosone/s/file?id=wjVg/PLOSOne_formatting_sample_main_body.pdf*](https://journals.plos.org/plosone/s/file?id=wjVg/PLOSOne_formatting_sample_main_body.pdf)

[*https://journals.plos.org/plosone/s/file?id=ba62/PLOSOne_formatting_sample_title_authorsaffiliations.pdf*](https://journals.plos.org/plosone/s/file?id=ba62/PLOSOne_formatting_sample_title_authorsaffiliations.pdf)

**Abstract**
The abstract section should be written according to the guidelines of the journal (Background, Methods, Results, Conclusion).

Please write keywords based on MeSH terms.

**Methodology**

In the " search strategy " section, Google Scholar is deleted; Because it is not a database but a search engine.

**Discussion**

I suggest you explain more how your study will help the health system and patients.
